# Supplementary material for: Enhanced Photocatalytic Hydrogen Production Activity by Constructing a Robust Organic-Inorganic Hybrid Material Based Fulvalene and TiO2
Source: Nanomaterials (Basel). 2022 Jun 3;12(11):1918. doi: 10.3390/nano12111918 (PMC9182102; doi:10.3390/nano12111918)
Supplement: Supplementary file 1 [file nanomaterials-12-01918-s001.zip › nanomaterials-1681999-supplementary.pdf]

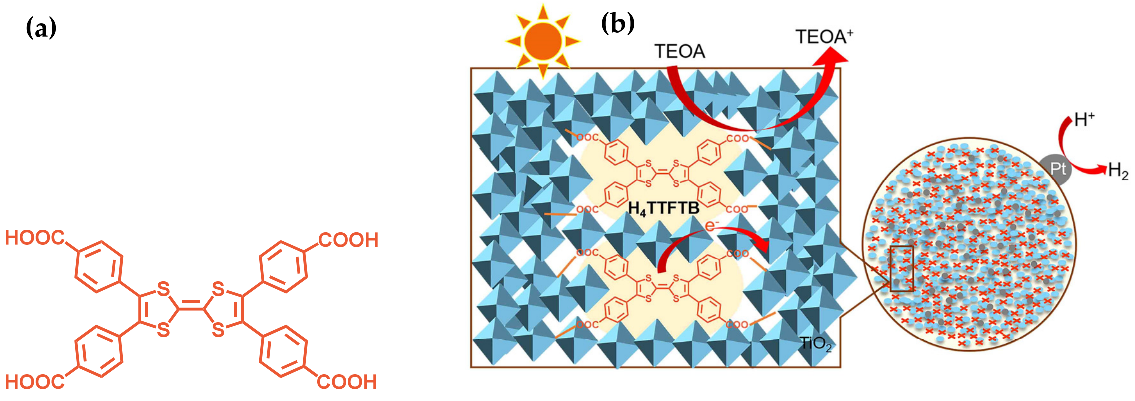

Figure S1. **(a)** The structure of H_4TTFTB, **(b)** illustration of the fabrication of the H_4TTFTB-TiO_2.

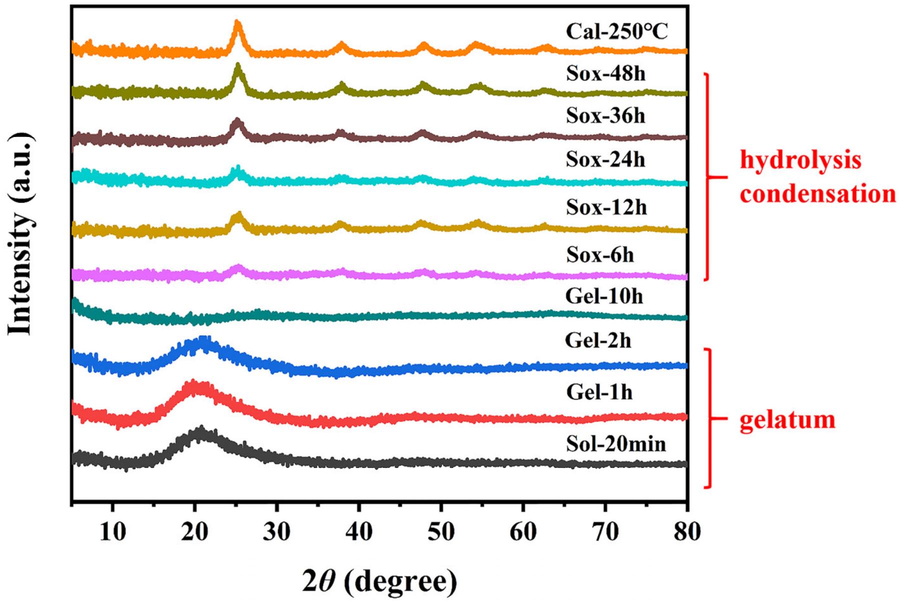

Figure S2. The XRD patterns of the intermediate products at different reaction times.

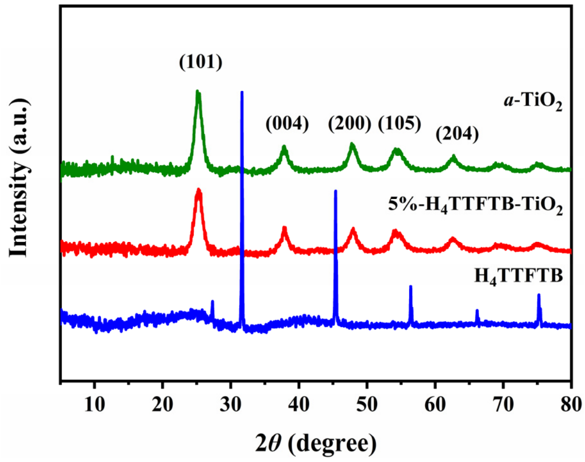

Figure S3. The XRD patterns of the *a*-TiO_2, H_4TTFTB and H_4TTFTB-TiO_2-5.0.

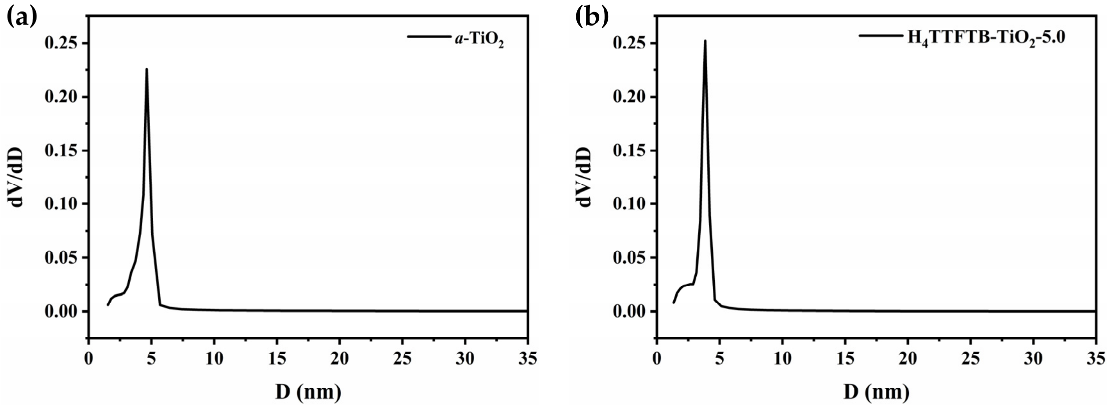

Figure S4. The pore size distributions of **(a)** *a*-TiO_2, **(b)** H_4TTFTB-TiO_2-5.0.

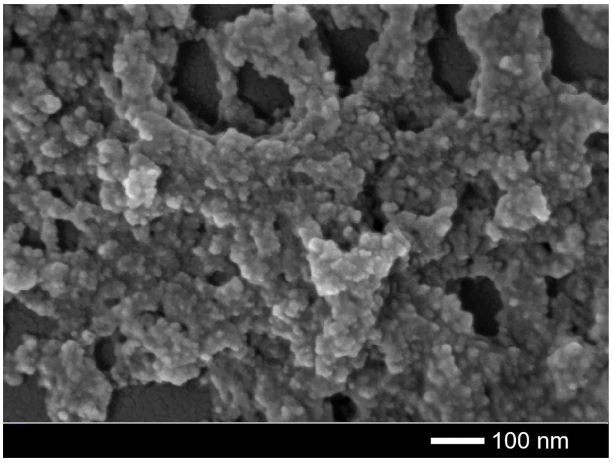

Figure S5. The SEM of H_4TTFTB-TiO_2-5.0.

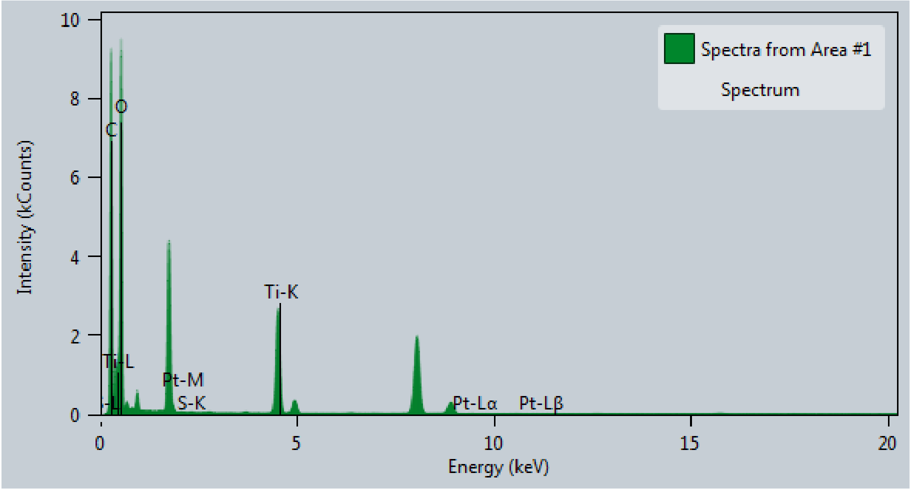

Figure S6. EDX of the Pt@H_4TTFTB-TiO_2-5.0.

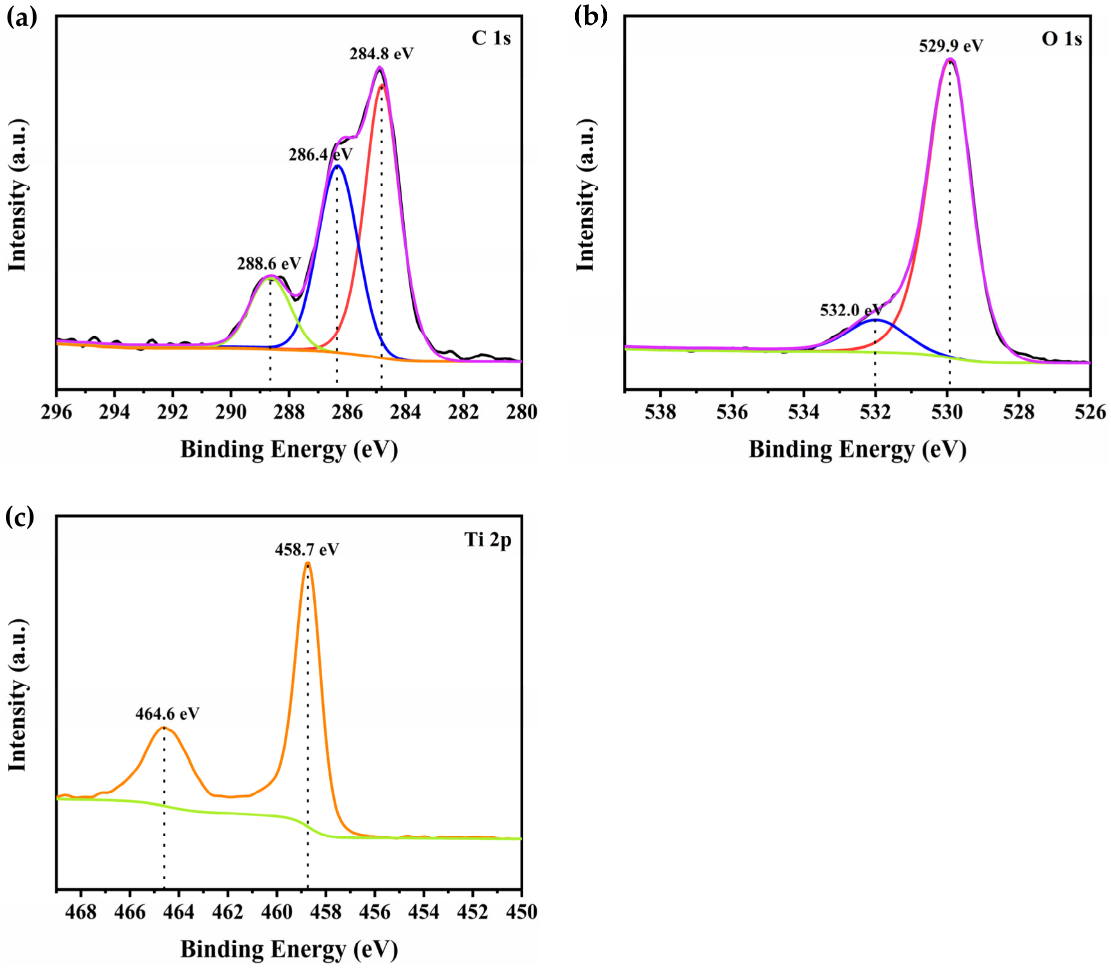

Figure S7. X-ray photoelectron spectra of *a*-TiO_2: (**a**) C 1s; (**b**) O 1s and (**c**) Ti 2p. Atomic ratio of Ti : O = 35% : 65% ≈ 1 : 2.

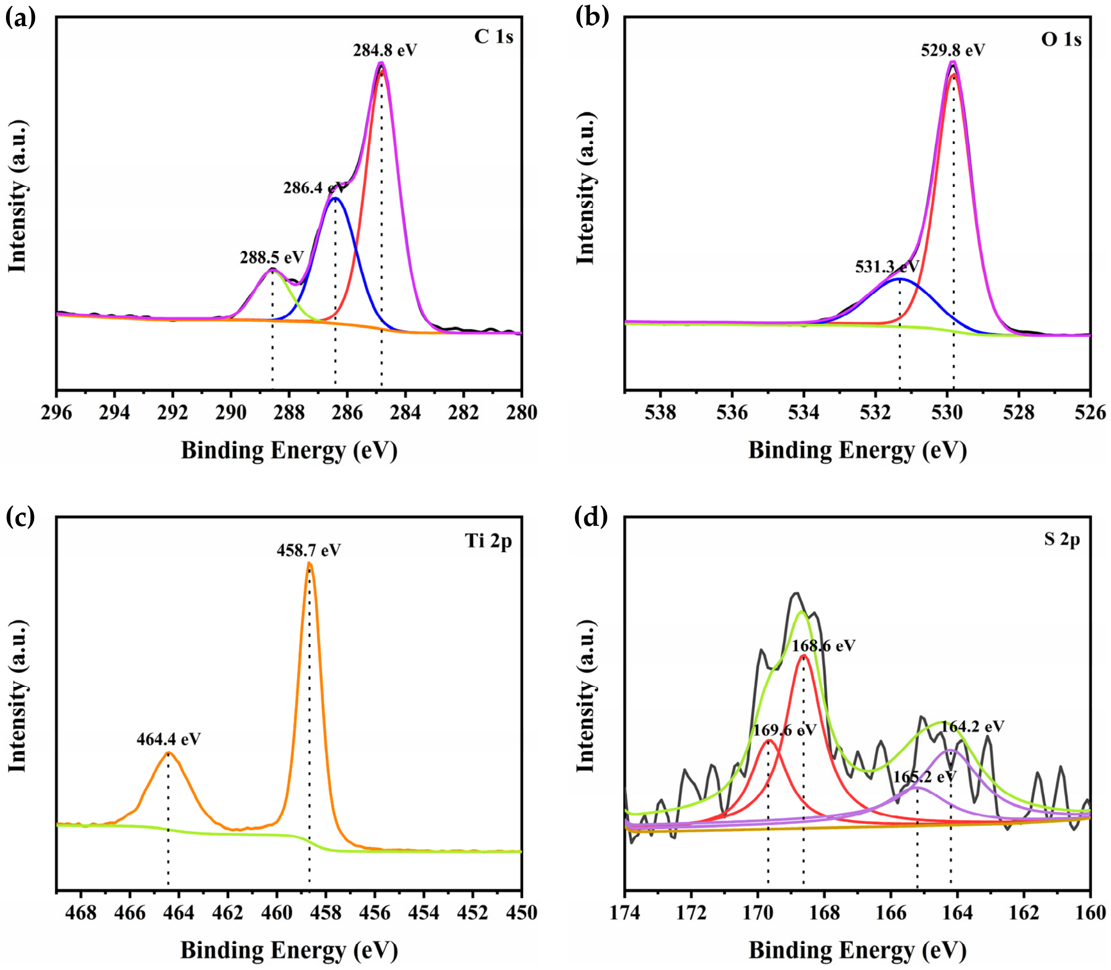

Figure S8. X-ray photoelectron spectra for of H_4TTFTB-TiO_2-5.0:(**a**) C 1s; (**b**) O 1s; (**c**) Ti 2p and (**d**) S 2p. Atomic ratio of Ti : O : C : S = 27% : 51% : 21% : 1.18%.

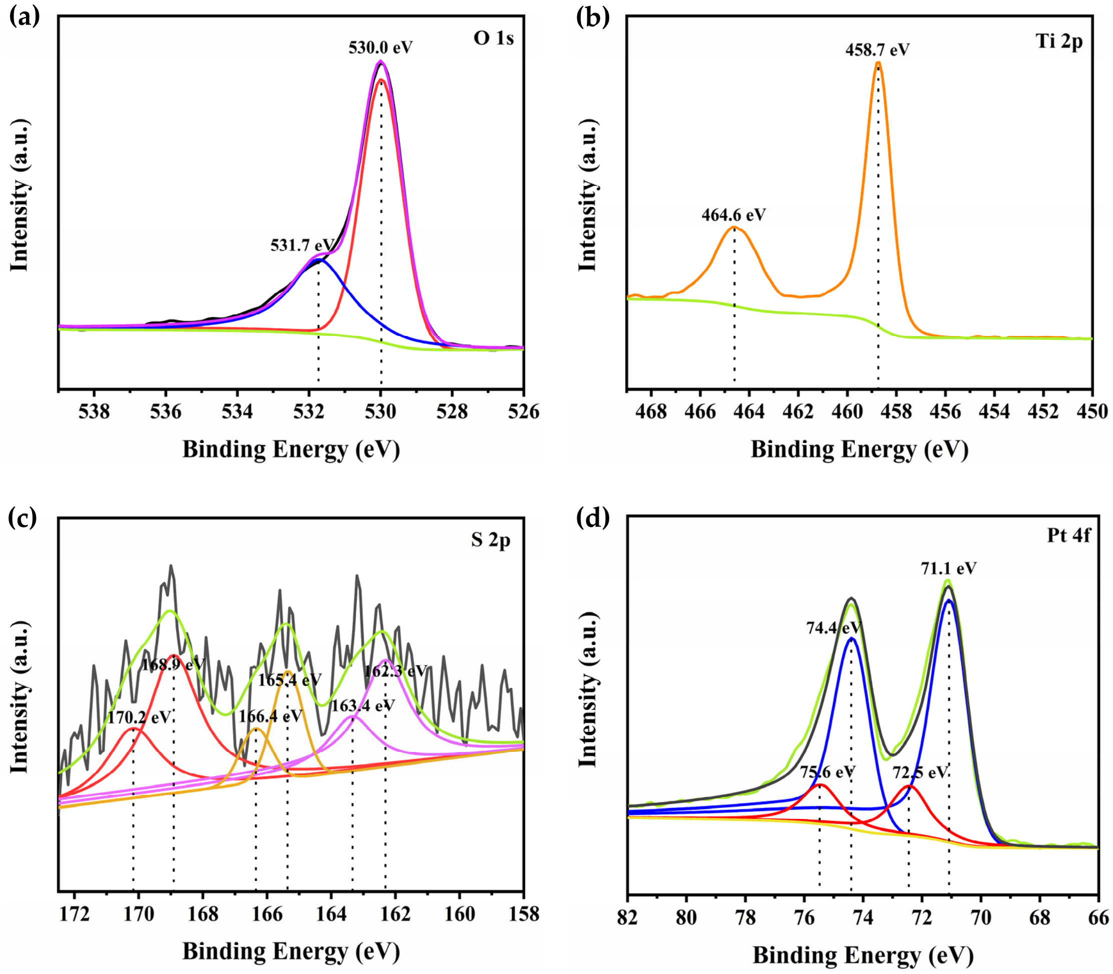

Figure S9. X-ray photoelectron spectra of of Pt@H_4TTFTB-TiO_2-5.0: (**a**) O 1s; (**b**) Ti 2p; (**c**) S 2p and (**d**) Pt 4f.

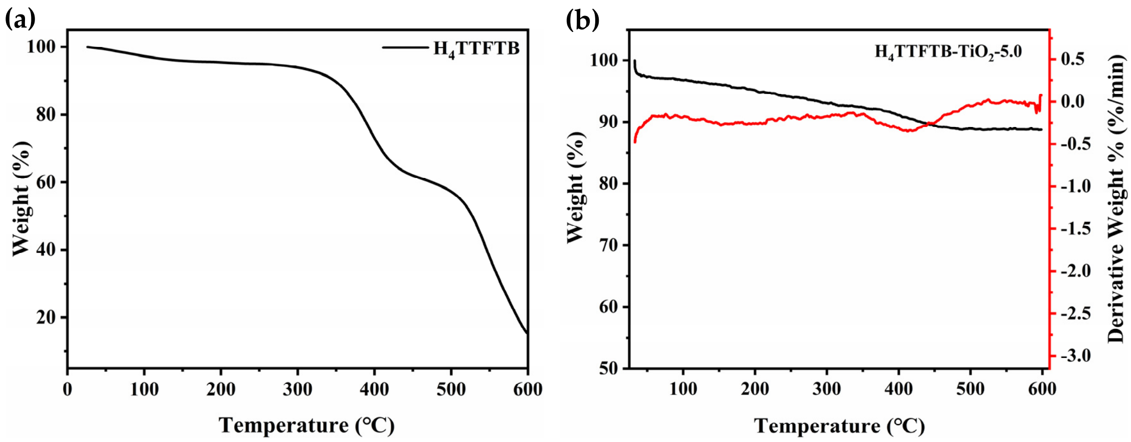

Figure S10. Thermogravimetry analysis of **(a)** H_4TTFTB compound，**(b)** H_4TTFTB-TiO_2-5.0.

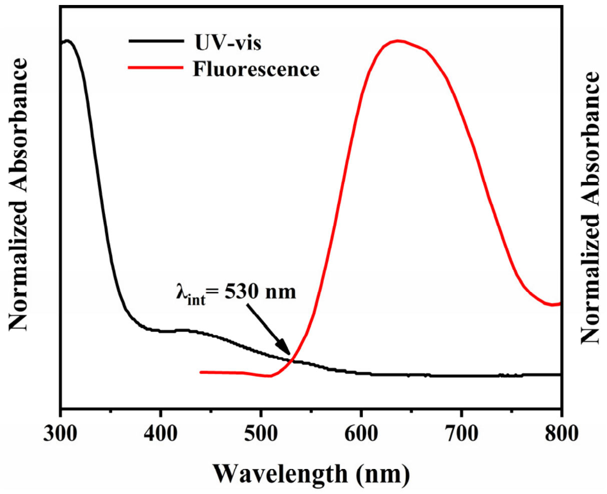

Figure S11. UV-vis absorption spectra and photoluminescence spectra of H_4TTFTB in DMF ($E_{0\text{-}0}$ = 1240/ λ int).

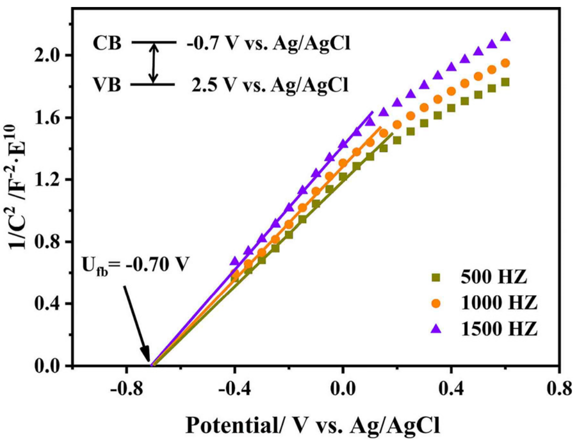

Figure S12. Mott-Schottky plot of *a*-TiO_2 in 0.5 M Na_2SO_4 aqueous solution.

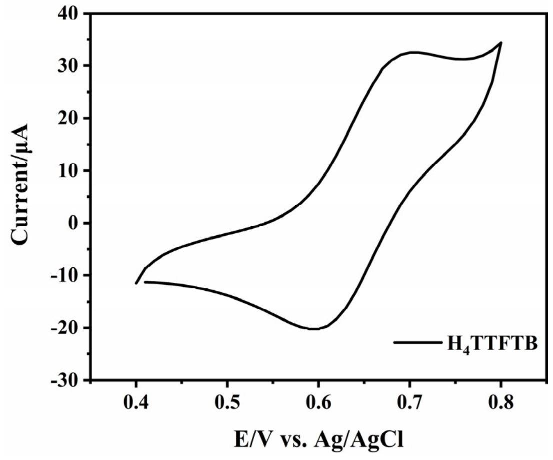

Figure S13. Cyclic voltammogram of H_4TTFTB in 0.1 M $TBAPF_6$ of DMF solutions measured with a scan rate of 50 mV s^{-1}.

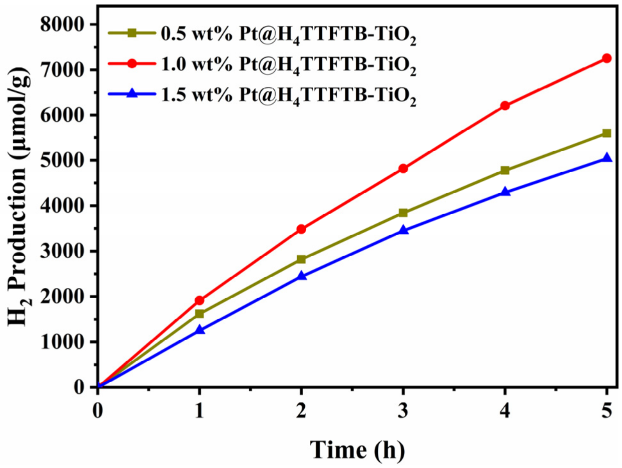

Figure S14. Comparison of photocatalytic H_2 evolution of 5 hours of H_4TTFTB-TiO_2-5.0 hybrid material under different loads of Pt.

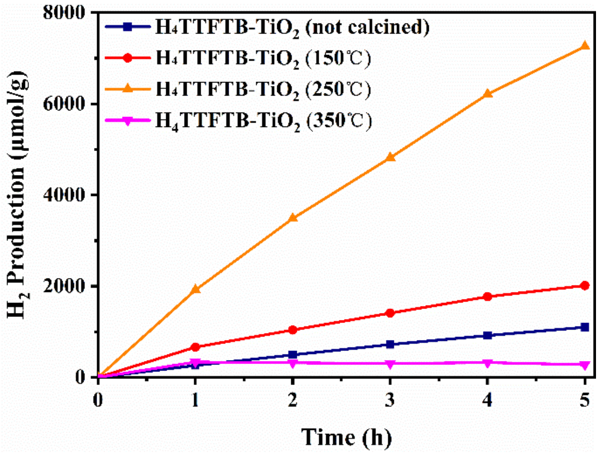

Figure S15. Comparison of photocatalytic H_2 evolution of H_4TTFTB-TiO_2-5.0 hybrid material under different calcined temperature loaded with 1.0 wt% Pt.

Table. S1. The amount of Pt NPs of Pt@H_4TTFTB-TiO_2.

Sample	Pt NPs Loading amount(wt%)
0.5 wt% Pt@H_4TTFTB-TiO_2	0.393
1.0 wt% Pt@H_4TTFTB-TiO_2	0.878
1.5 wt% Pt@H_4TTFTB-TiO_2	1.288

Table. S2. BET surface area, pore volume and pore size of *a*-TiO_2, H_4TTFTB-TiO_2-5.0 derived from 77 K N_2 sorption isother.

Sample	Surface area (m^2/g)	Pore size (nm)	Pore volume (cm^3/g)
a-TiO_2	184.10	4.411	0.257
H_4TTFTB-TiO_2-5.0	212.65	3.630	0.239

Table. S3. Atmoic Fraction of Pt@H_4TTFTB-TiO_2.

Z	Element	Family	Atomic Fraction (%)	Atomic Error (%)	Mass Fraction (%)	Mass Error (%)	Fit Error (%)
6	C	K	58.16	7.63	43.36	3.48	2.79
8	O	K	34.35	8.16	34.12	7.29	0.55
16	S	K	0.01	0.00	0.02	0.01	14.70
22	Ti	K	7.46	1.38	22.16	3.38	0.04
78	Pt	L	0.03	0.00	0.34	0.05	2.28

Table S4. Comparison of H_2 evolution activity of TTF and TiO_2 photocatalytic systems.

Catalyst	Co-catalyst	Sacrificial agent	Solvent	Light source (nm)	H_2 Production rate (μ mol h^{-1} g^{-1})	Ref
H_4TTFTB-TiO_2	Pt	TEOA	H_2O	＞400	1452	This work
Zn-TPY-TTF	Pt	TEA	H_2O	＞400	14727	[45]
G3T3	Pt	TEOA	H_2O	＞400	24560	[19]
DPPCN/TiO_2	Pt	TEOA	H_2O	＞400	12080	[18]
DPPCA/TiO_2	Pt	TEOA	H_2O	＞400	8400	[18]
MOC-16/TTF	Pd	TEOA	DMSO-H_2O	＞420	7344.9	[46]
Rh B-Co/TiO_2	Co	-	water	＞400	227.3	[47]
P42-TiO_2	Pt	TEOA	H_2O	＞420	745.0	[48]
PI-OMe-TiO_2	Pt	TEOA	H_2O	＞420	1190	[26]
3C/TiO_2	Pt	TEOA	H_2O	＞420	247	[49]
ZnPc/TiO_2	Pt	TEOA	H_2O	＞420	3448	[22]
CoPc/TiO_2	Pt	TEOA	H_2O	＞420	3328	[22]
B-Car/TiO_2	Pt	ascorbic acid	Water	＞420	249	[50]
PAN-Pt@TiO_2	Pt	thioglycolic acid	H_2O	＞420	61.8	[51]
Chl-3-TiO_2	Pt	ascorbic acid	water	＞400+ ＞600	263.3	[52]
Eosin Y^+ $Ni(OH)_2/TiO_2$	$Ni(OH)_2$	TEOA	H_2O	＞420	1576	[53]
MZ-341/TiO_2	Cu_2WS_4	TEOA	water	＞420	1406	[54]
1-P25	Pt	TEOA	water	＞400	2364	[55]
FL@MOC-PC6	Pd^{2+}	TEOA	water	＞420	2402	[56]
